# Molecular Dynamics Study of an Amorphous Polyethylene/Silica Interface with Shear Tests

**DOI:** 10.3390/ma11060929

**Published:** 2018-05-31

**Authors:** Xiaoying Zhuang, Shuai Zhou

**Affiliations:** 1 Institute of Continuum Mechanics, Leibniz University Hannover, 30167 Hannover, Germany; zhuang@ikm.uni-hannover.de; 2 Department of Geotechnical Engineering, Tongji University, Shanghai 200092, China; 3 Institute of Structural Mechanics, Bauhaus Universitat-Weimar, D-99423 Weimar, Germany

**Keywords:** polyethylene, damage process, composite, silane coupling agent, interface

## Abstract

An amorphous polyethylene/silica (PE/S) interface exists in many materials. However, the research of the interfacial properties at microscale is lacking. Shear failure and adhesion properties of an amorphous PE/S interface are studied by molecular dynamics. The effects of PE chain length, the number of chains, and coupling agents on the shear behavior and interfacial adhesion are investigated. It is found that the modified silica (mS) surface induces an increase in the adhesion strength compared to unmodified S. The damage process and failure mode of the PE/S and PE/mS interface are analyzed at microscale. The contribution of bond length, bond angle, torsional potentials, and nonbonded energy is estimated as a function of the shear deformation to clarify the deformation mechanisms. The energy partitioning results indicate that the elastic, yield, and postyielding regions are mostly controlled by the nonbonded interactions. The dihedral motions of the chains also have an influence. Furthermore, the simulation results exhibit how the internal mechanism evolves with the shear deformation.

## 1. Introduction

Organic-inorganic interface exists in many material systems that can be found broadly in natural and synthetic materials, such as polyethylene/silica (PE/S) interfaces that are found in composites [1]. Based on prior experimental and numerical research on interfacial properties of bonded systems [2,3,4,5,6,7,8], it is known that the structural and mechanical integrity of the interface is highly affected by the physical and chemical interactions between the interface and the surrounding region at the microscale. Hence, a clearer knowledge of the PE/S interfacial properties is important.

Molecular dynamics (MD) is an effective method to study the microscopic properties of a material. MD simulation is based on the modelling at an atomistic level. The smallest unit in the atomistic model is an atom, which can provide natural mechanisms of a phenomenon. In particular, the integrity of the bonded material system can be studied from a fundamental perspective by monitoring the interactions between two materials along the interfacial region at a molecular level by MD. Recently, some researchers used it to study the properties of bi-material systems, such as the chitin/protein interface [9], polyethylene/graphene interface [10], epoxy/S interface [11], carbon fiber reinforced polymers/wood interface [12], polyimides/S glass interface [13], hexagonal boron nitride/polyethylene interface [14], dihydroxyphenylalanine/S interface [15], S/polystyrene interface [16], carbon nanotubes/epoxy interface [17], polyvinylidene fluoride binder/copper interface [18], epoxy/copper interface [19], etc. The tensile strength, shear strength, effects of strain rate, effects of cell size, effects of conversion, effects of temperature, interaction bonding energy, effects of entanglement, failure mode, confined condition, density profile, and failure strain have been extensively investigated. The mechanisms of the above mentioned interfaces are explored at the microscale. Nevertheless, the shear fracture behavior of PE/S interface at microscale has not been investigated.

It is well known that coupling agents [20] and surfactant [21] can enhance molecular bonding between the polymer matrix and inorganic particulates, and hence strengthen the interfacial adhesion. However, selecting the appropriate coupling agent for specific composite system can be a time-consuming process in terms of experimental study. MD simulations may be adopted to reduce the number of trial-and-error steps in identifying the coupling agent and therefore significantly reduce the time for coupling agent selections. Though the suitable treatment of the material surface for enhancing the interactions and bonding to other materials surface has been explored by MD previously [20,22], the PE/S interface system has not been considered.

In this research work, the adhesion between PE and neat S or mS with silane coupling agents is evaluated via MD simulations. The shear simulations are performed on the interface models. The shear stress versus displacement response is obtained through stretching the top layer of the PE during the simulation. The effect of chain length and the number of the polymer chains, and the modified S by coupling agents on the interfacial adhesion is investigated. The evolution of energy of the polymer chains during the shear process is analyzed to comprehend the shear behavior. Internal mechanisms that are associated with chain movement are also evaluated during the process to study the interface behavior.

## 2. Simulation Details

### 2.1. Model Construction

The Material Studio is used to produce the atomistic model and the obtained structure is relaxed using the Large-scale Atomic/Molecular Massively Parallel Simulator (LAMMPS) distributed by Sandia National Laboratories (Livermore, CA, USA) [23]. There are different types of potentials (e.g., Consistent Valence Forcefield (CVFF), polymer consistent force field (PCFF), Dreiding, etc.) in simulating the interactions between atoms and they are suitable for different cases. In order to obtain the interactions between atoms in the present system, the Dreiding potential is utilized in PE, S, and the coupling agents [24], since it has been parameterized and validated for both organic and inorganic materials [25,26,27]. The bonds and angles are treated as harmonic. The van der Waals terms are treated as Lennard-Jones 12-6, and the charges are ignored [24]. Related values of the parameters in the Dreiding potential come from previous research [24].

#### 2.1.1. PE Model

In order to investigate the effect of chain length and the number of the PE chains (i.e., (C_2_H_4_)_n_) on the simulation results, three types of PE cells with different combinations of the chain length and the number of chains are considered. The entanglement length of PE chains is in the range of 6090–carbon atoms [10]. Therefore, 150 carbon atoms around twice the entanglement length [22] are considered to investigate the effect of entanglements on the shear simulations of the interfaces. Two types of PE models with 76 and 150 carbons on the backbone of a chain are used for comparison. In each type of the PE model, 20 and 40 chains are considered to study the effect of the chain number on the adhesion properties and shear strength.

The Verlet velocity algorithm is utilized for integration with 1 fs time step in the simulations with 12 Å cutoff distance. Potential energies of the PE boxes are minimized using following multistep procedure. At first, an initial energy minimization is performed on the PE box using the Conjugate Gradient algorithm with the tolerance of 10^−9^. Then, a Nose-Hoover thermostat [28] is applied for 100 ps (∆t = 1 fs) at 600 K, followed by a Nose-Hoover barostat [29] at atmospheric pressure for 1 ns at the same temperature. The next relaxation cools the structure down to 300 K for 1.5 ns followed by further relaxation of 1 ns at 300 K to take the structure to its equilibrium density [25]. The densities of the final equilibrated periodic PE boxes are in the range of 0.78–0.85 g/cm^3^, which are in agreement with the experimental data reported previously [22].

**Figure 1:**
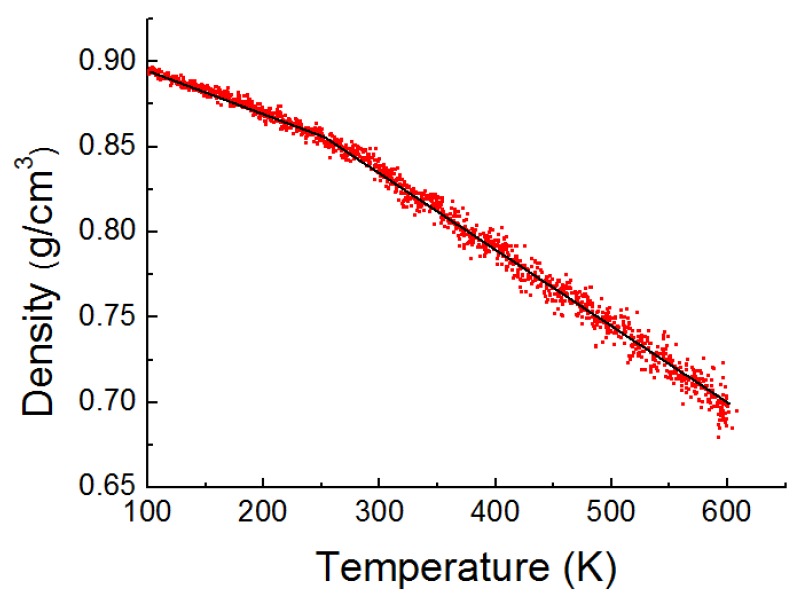
Temperature dependence density of the PE(150–20)/S model.

The glass transition temperature (Tg) is determined from the density changes during cooling of the PE systems [25]. It can be determined from the change in slope of polymer density versus temperature curve. In our simulations, the systems are relaxed at 600 K and are then cooled down to 100 K for 2.5 ns with a cooling rate of 0.2 K/ps [25]. The glass transition temperature Tg is identified as the turning point with fitted lines of the MD density-temperature data. Figure 1 indicates the density variation versus temperature for the PE model containing 20 PE chains, each one with 150 carbon atoms. The glass transition temperature Tg = 252 K and the density *ρ* = 0.83 g/cm^3^ at 300 K when the PE model consists of 20 chains, each chain with 150 carbon atoms. The calculated Tg values for the models with two different chain lengths (i.e., 76 and 150) and two different chain numbers (i.e., 20 and 40) are in the range of 232 K–264 K. The calculated Tg values are in the experimental temperature range of 190–300 K, as reported previously in other research works [30,31]. In this study, an agreement between the calculated physical properties of the PE models, i.e., density and Tg value, and the experimental results indicates that the stable and equilibrated PE models are achieved.

#### 2.1.2. S Model

**Figure 2:**
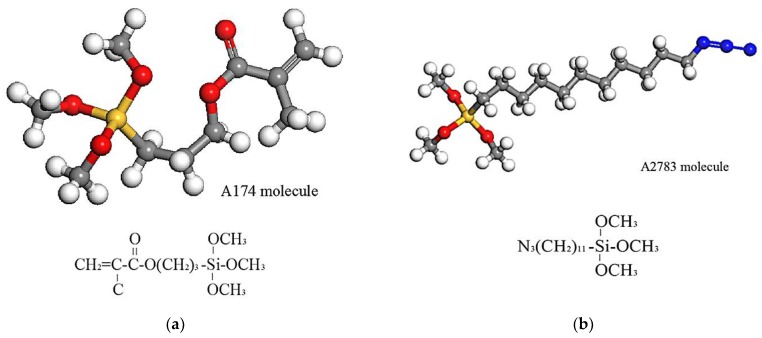
The chemical structure of (**a**) A174; and (**b**) A2783.

There are many kinds of S coupling agents. A174 [32,33] and A2783 [34] can be used as the S coupling agent. A174 refers to γ-methacryloxypropyl trimethoxy silane. A2783 belongs to azidofunctional trialkoxysilane. The chemical structures of A174 and A2783 are exhibited in Figure 2. The coupling mechanism is explained in detail previously [34,35,36]. Here, those two kinds of coupling agents are considered in our simulations to study the interfacial properties.

**Figure 3:**
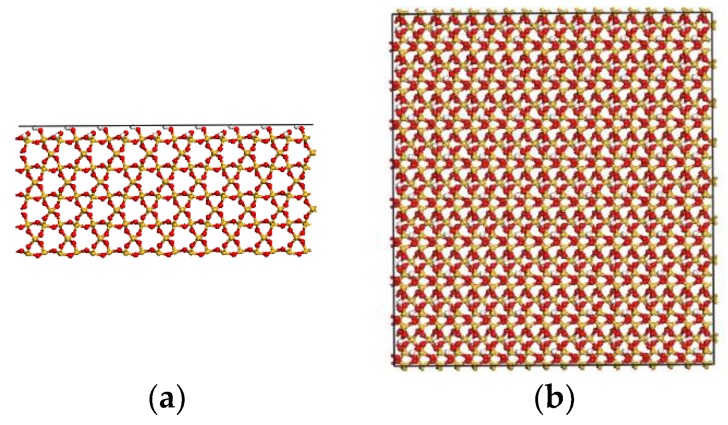
(**a**) Side view of S (100) surface; and (**b**) top view of S (100) surface.

The neat S and surface-modified S with A174 and A2783 are used as the single sheet substrate. The S (100) surface is studied [11]. For the S substrate, hydrogen atoms are used as termination atoms at its upper surface [11]. The side and top view of S with the (100) surface is illustrated in Figure 3.

**Figure 4:**
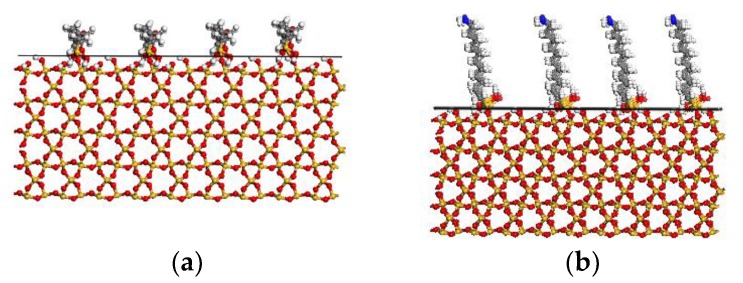
(**a**) Side view of A174-modified S; and, (**b**) side view of A2783-modified S.

According to previous research, a horizontal spacing between different coupling agent around 0.5 nm is adopted [20]. On the modified surface, the A174 is assumed to attach onto only one surface of the S via chemical covalent bonding [20]. Figure 4a illustrates S (100) with the coupling agent A174. The A2783 attaches onto both S and PE via chemical covalent bonding [34]. Figure 4b shows S (100) with the coupling agent A2783. An energy minimization run of mS is carried out using a Conjugate Gradient algorithm [25].

#### 2.1.3. Interface Model

**Figure 5:**
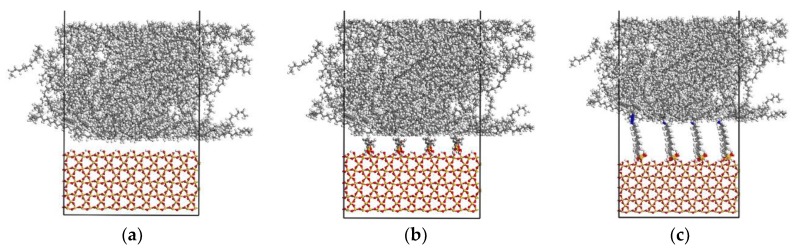
The molecular model of (**a**) PE(76–40)/S; (**b**) PE(76–40)/mS1; and, (**c**) PE(76–40)/mS2.

To build an interface model, the S or mS is introduced to the PE surface at the distance (i.e., 0.5 nm) less than the cutoff radius. The simulated polymer box is further enlarged to 1100 Å in the normal direction to assure that the adjoining systems do not interact in this direction during the interface creation and elongation when the three-dimensional (3D) periodic boundary conditions are applied [10]. First, the resultant system is heated up to 500 K for 500 ps and then equilibrated at 300 K with a cooling rate of 0.2 K/ps. Next, it is equilibrated at 300 K for further 15 ns using the constant temperature, constant volume (NVT) ensemble. The equilibrated interfaces, including PE chains over the neat S, A174-modified S, and A2783-modified S surfaces are designated as PE(x–y)/S, PE(x–y)/mS1, and PE(x–y)/mS2, respectively. Here, x is the chain length and y is the number of chains. Figure 5 illustrates the constructed PE(76–40)/S, PE(76–40)/mS1, and PE(76–40)/mS2 interface models.

### 2.2. Simulation of Sliding Deformation of the Interfaces

**Figure 6:**
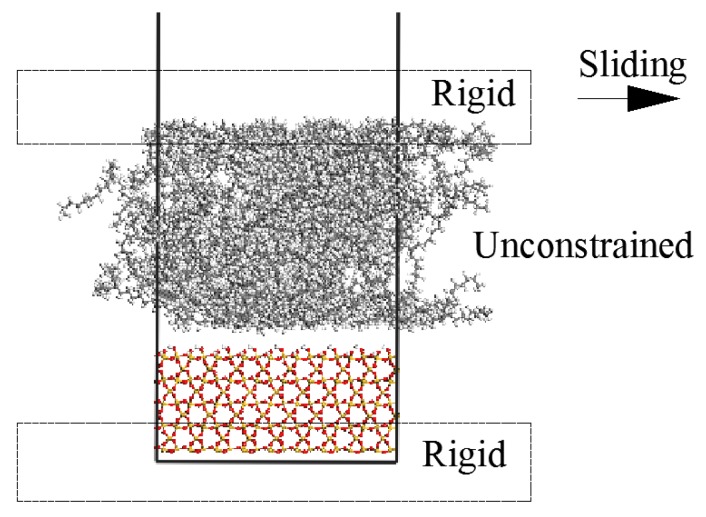
The representation of PE(76–40)/S interface model with the imposed rigid parts in the shear process.

For simulation of PE/S interface deformation, the atoms of the free polymer top layer, as well as the bottom layer of S are kept frozen while leaving the middle part of PE and coupling agents unconstrained. The top frozen layer of the PE moves with a prescribed velocity to apply the horizontal interface deformation in the shear case (Figure 6). In shear simulation, the separation is carried out by the relative movement of the phases in the direction parallel to the interface. The separation is performed in steps, where the rigid top layer of PE is displaced by 1.0 Å at each step. The bottom layer of S is fixed during the process. At each displacement step, the middle unconstrained part of the interface is allowed to dynamically equilibrate for 1 ns using a NVT simulation. This deformation process is continued until the final failure of the interface occurs. Displacement of 1.0 Å in 1 ns is equal to a rate of 0.1 m/s^−1^, a high separation rate when compared with those that were used in standard laboratory tests [10]. The shear stress is calculated using the virial theorem [37] by considering the kinetic and potential energy of all the particles throughout the middle unconstrained PE zone. The stress in each step of deformation is determined by time-average over the latter half of the equilibrium interval and recorded versus displacement to show stress-displacement curves.

## 3. Results and Discussion

### 3.1. Stress-Displacement Behavior

**Figure 7:**
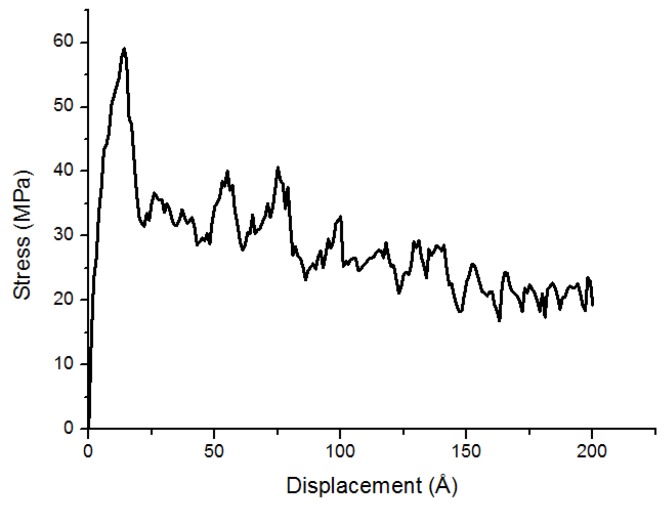
The shear stress-displacement curve of the PE(76–40)/S interface.

The shear stress-displacement curve of PE(76–40)/S deformed at 300 K is shown in Figure 7. The curve at the low displacement or elastic region is ascending. After a maximum stress at the yield point, the stress decreases due to the change of the configuration of chains and chain slipping. Finally, the shear stress fluctuates at the end of the simulation. The MD simulations capture the detailed fracture process at the atomic scale.

**Figure 8:**
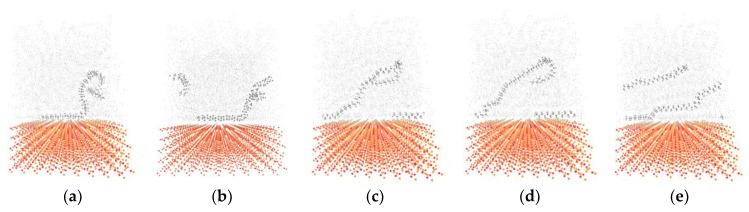
Snapshots of shear simulation of PE(76–40)/S interface at various displacements: (**a**) 2 Å, (**b**) 14 Å, (**c**) 50 Å, (**d**) 100 Å, and (**e**) 200 Å.

Figure 8 indicates the snapshots of PE(76–40)/S interface at different deformation process stages at 300 K. One chain is chosen to illustrate the movement of the whole PE structure in Figure 8. Other PE molecules are zoomed out to highlight the target chains. During the increase of the shear traction, the PE chains are stretched rightwards. As the normal displacement is fixed, no void appears in the PE. The stress drop that is shown in Figure 7 around the shear displacements of 14 Å is due to the separation among the PE chains and the S surface. The first snapshot (Figure 8a) indicates the interface near the elastic region, where no visible damage is observed. The target chain is bending and almost does not move. There is a rightward move of the target chain at the maximal shear stress in Figure 8b. The post-yielding behavior is exhibited by debonding between the chains, chain slipping, and disentanglement extending to an ultimate failure mode. The chain movement is witnessed in Figure 8c–e. In the middle of the simulation, the entire chain lies almost horizontally near the surface, while the top part of it is still bending (Figure 8d). With the shear load, the bending chain becomes totally straightened and slipping on the surface of S (Figure 8e). The chain then continuously translates until the end of the simulation, keeping the horizontally stretched manner. The chain moves slowly at first and then moves faster. The PE chains move rightwards in the shear loading direction. They go across the right boundary and then move into the simulation box from the left side due to the periodic boundary condition. At the same time, the chains are slipping against each other in the regions of the polymer chains with weaker intermolecular interactions and less entanglement. The failure position is at the PE/S interface. The behavior of the polymer chains may considerably change at the interface in the shear case in comparison to that in the tensile case [10]. The voids exist in the bulk of PE in the tensile case, while they do not occur in the shear case [10]. The polymer chains are being pulled out from their starting configurations. The separation is caused by the weak interaction of the van der Waals interactions acting at the interface. The breakdown of nonbonded interactions between the PE and S surface results in the adhesive failure mode.

### 3.2. Effect of Chain Length, Number of Chains and Coupling Agents

**Figure 9:**
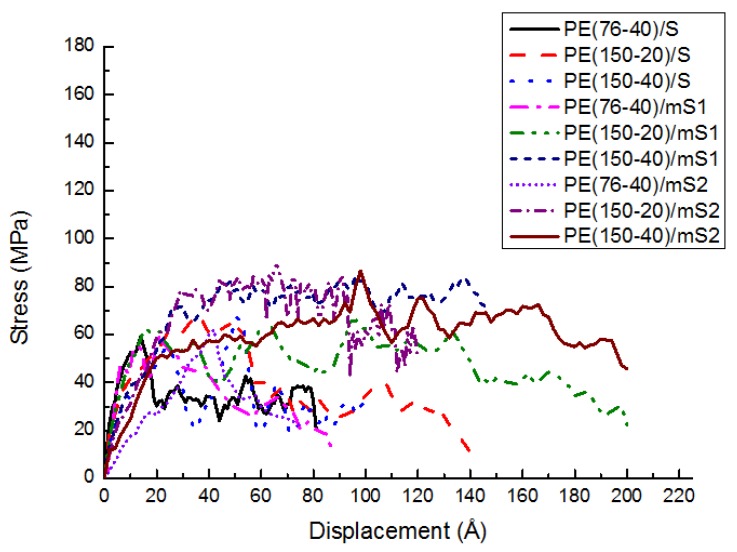
The effect of PE chain length, number and coupling agent on the stress-displacement behavior of PE/S at 300 K.

In the sliding mode, the shear stress is calculated while the fixed top layer is moved horizontally. Figure 9 shows the shear response for different cases in the sliding mode. The shear response presents a similar trend to the normal traction-separation response [10]. Initially, the shear stress increases. The stress then reaches the maximum. After the peak, the stress gradually decreases. Large fluctuation exists since the displacement in the normal direction is fixed.

The maximum stress and corresponding displacement are exhibited in Table 1. The shear strengths of PE(76–40)/S, PE(150–20)/S, and PE(150–40)/S are 59.3 MPa, 66.8 MPa, and 67.4 MPa, respectively. The corresponding displacements at the maximal stress are 14 Å, 34 Å, and 50 Å, respectively. It illustrates that with a higher chain length and number, the shear strength and the corresponding displacement increase. It is caused by the stronger entanglement. The stress transferring path is more complex, which induces a larger stress and displacement. From Table 1, the shear strength of modified S is greater than that of unmodified S. Further, the shear strength of the A2783-modified S is greater than that of the A174-modified S. The reason is that the PE can interact with A174 and A2783 better than the S. A2783 that is connected with PE by chemical bonds, which are stronger than the nonbond interactions in A174. The improvement of the shear strength of PE(76–40) by coupling agents is not obvious due to the weak matrix, even though the interface has been strengthened. The PE(150–40)/mS1 and PE(150–40)/mS2 prefer the cohesive failure mode, while the PE(150–40)/S tends to fail in the adhesive failure mode. Hence, the difference between PE(150–40)/mS2 and PE(150–40)/mS1 is small, while the difference between PE(150–40)/S and PE(150–40)/mS1 is large. The A2783-modified S has the maximum displacement at the maximum shear stress, since the A2783 molecule is longer than the A174 molecule.

**Figure 10:**
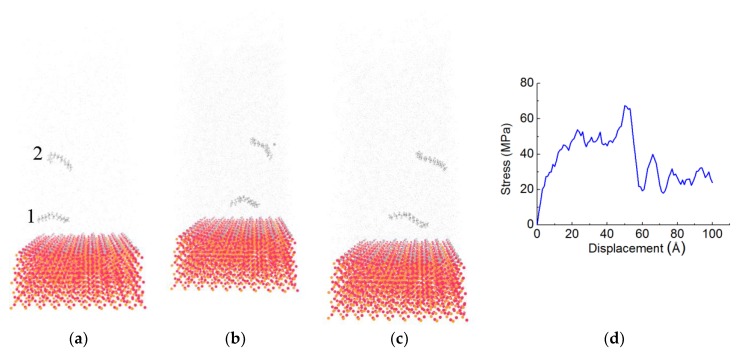
Snapshots of shear simulation of PE(150–40)/S interface at various displacements: (**a**) 2 Å, (**b**) 50 Å, (**c**) 100 Å, and (**d**) the stress curve.

Figure 10 displays representative snapshots of chains in the PE(150–40)/S. The relevant stress curve of PE(150–40)/S is presented in Figure 10d. One chain (i.e., chain 1) that is shown in Figure 10a slides near the S surface as the shear displacements are applied. The other one is in the middle of the PE (i.e., chain 2). After a shear displacement, both chains begin moving. Their configuration does not change during the simulation. Both chains then continuously translate until the end of the simulation, keeping the horizontally stretched manner (Figure 10c). From the snapshots, we can confirm that, in the sliding mode, the PE is separated at the interface, as shown similarly in the PE(76–40)/S. The averaged shear stress at the end of the simulation is determined by the sliding motion at the interface. Different chain length and the number of chains do not influence the failure mode.

**Figure 11:**
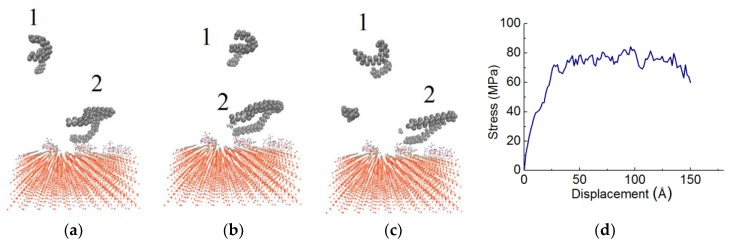
Snapshots of shear simulation of PE(150–40)/mS1 interface at various displacements: (**a**) 2 Å, (**b**) 50 Å, (**c**) 100 Å, and (**d**) the stress curve.

**Figure 12:**
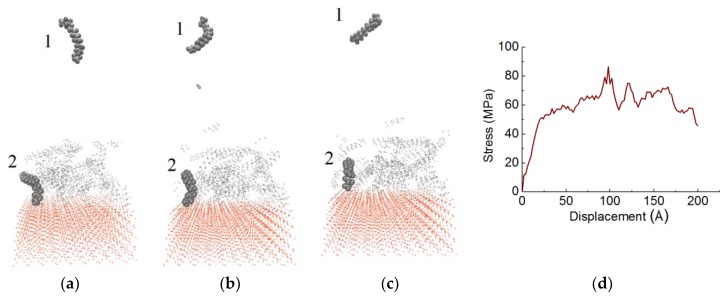
Snapshots of shear simulation of PE(150–40)/mS2 interface at various displacements: (**a**) 2 Å, (**b**) 100 Å, (**c**) 200 Å, (**d**) the stress curve.

The influence of coupling agents is illustrated in Figure 11 and Figure 12. The upper polymer chain (i.e., chain 1) shown in Figure 11 slides in the middle of the PE. The other one is at the bottom of the PE (i.e., chain 2). The relevant stress curve of PE(150–40)/mS1 is displayed in Figure 11d. Although the upper chain is stretched horizontally, the bottom chain is adhered to the substrate and it does not move at first. After a shear displacement of 50 Å, the bottom chain begins moving. The top chain moves quickly, while the bottom chain moves slowly. The bottom chain is entangled with the A174 coupling agent. Hence, the speed is slowed down. From the snapshots, the PE is separated in the middle since each chain in Figure 11 belongs to the top and bottom of the PE, respectively. The bottom chain is almost static and the top chain moves with the sliding strains. As illustrated, the chain segments in the middle layer are easily stretched and pulled out. After the cohesive failure, the PE separates into two parts, including an upper separated layer and a thin layer remaining in close contact with the S surface. In the interphase with longer chains, the connection between these two regions should be stronger than the interphase with shorter chains in Figure 8 due to a higher entanglement density. However, the cohesive failure mode happens. It can be attributed to the weak polymer layer as compared with the modified interface. Accordingly, the failure happens inside the weaker bulk region.

The shear simulation of PE(150–40)/mS2 interface is depicted in Figure 12. The relevant stress curve of PE(150–40)/mS2 is exhibited in Figure 12d. Some chains are chosen to exhibit the deformation of the interface. The upper polymer chain (i.e., chain 1) that is shown in Figure 12 slides in the middle of the PE. The other one is at the bottom of the PE (i.e., chain 2). First, as the shear strain increases, the chains do not move. They swing around their initial position. Then, many chains in the PE slide and the shear stress begins to decrease. Although chain 1 is stretched horizontally, chain 2 is adhered to the substrate and does not move (Figure 12b). The bottom chains are entangled with the A2783 coupling agent by chemical bonds. Hence, no chain moves at the interface. In the middle part of the PE bulk, the chains are straightened and inclined. From Figure 12, the PE is separated in the middle, as displayed similarly in the PE(150–40)/mS1. When compared with PE(150–40)/S and PE(150–40)/mS1, the bottom chains in PE(150–40)/mS2 never move due to the covalent bond between PE and A2783. The PE/mS1 and PE/mS2 prefer the cohesive failure mode, while the PE/S tends to fail in the adhesive failure mode. The modified S surface causes an increase in the adhesion strength as compared with unmodified S due to the PE chains adhered to the modified S by the coupling agent. The actual shear thickness of PE(150–40)/mS2 is thicker than that of PE(150–40)/mS1 since the A2783 molecule is longer than the A174 molecule. Hence, the shear strength of PE(150–40)/mS2 is greater than that of PE(150–40)/mS1.

### 3.3. Potential Energy Evolutions

**Figure 13:**
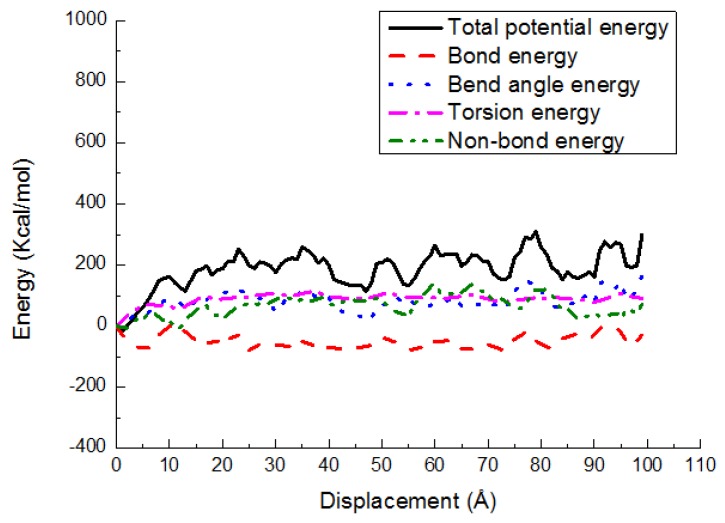
Potential energy-displacement curves for the PE(150–40)/S interface in the shear case.

**Figure 14:**
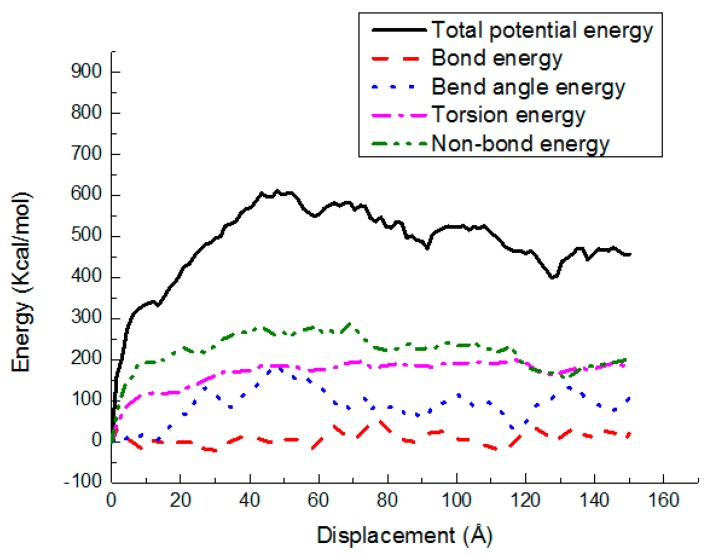
Potential energy-displacement curves for the PE(150–40)/mS1 interface in the shear case.

**Figure 15:**
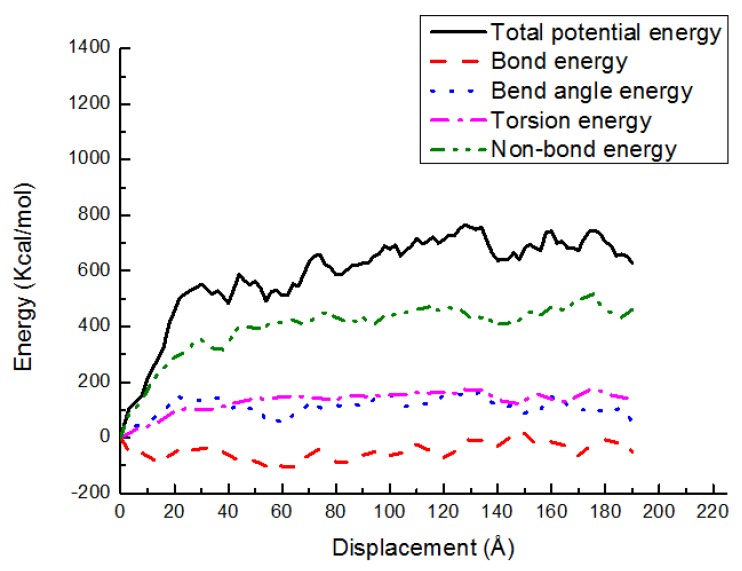
Potential energy-displacement curves for the PE(150–40)/mS2 interface in the shear case.

The energy changes are exhibited in various potential energy contributions, such as bond stretching, angle bending, torsion, and nonbond energy, versus shear displacement for the PE(150–40)/S, PE(150–40)/mS1, and PE(150–40)/mS2 interfaces at 300 K in Figure 13, Figure 14 and Figure 15, respectively. As shown, the deformation is performed via the bond angle and dihedral torsion instead of the direct bond stretching because of their significant changes during the deformation of the above-mentioned interface systems. The bond energy experiences a negligible change, illustrating that the bond breakage does not happen during the shear simulation. The shear deformation leads to the increase of the torsion energy and the bend angle energy. The rising of them implies that the angles and dihedral angles are changed from their equilibrium state and more distorted during the deformation process. It can also be observed that the nonbonded energy does not increase greatly due to the normal position is fixed and the PE does not leave the interface. The nonbond interactions of PE(150–40)/mS1 and PE(150–40)/mS2 interfaces are greater than that of PE(150–40)/S, which implies that the coupling agent can increase the interfacial actions in the shear case. Additionally, the dihedral energy and bend angle energy play a significant role in the postyielding regime of the PE(150–40)/S interface, including plastic deformation and chain disentanglement. It can be observed that in the shear deformation of equilibrated PE chains either in the pure bulk or at the vicinity of the S, the nonbonded energy plays the crucial role of the polymer.

**Figure 16:**
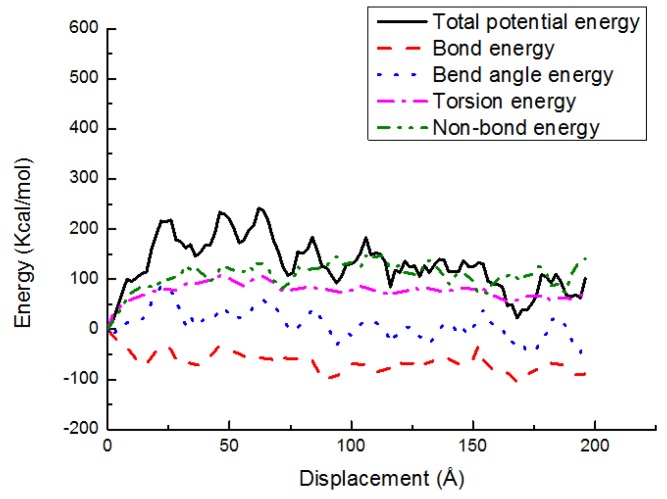
Potential energy-displacement curves for the PE(150–20)/S interface in the shear case.

**Figure 17:**
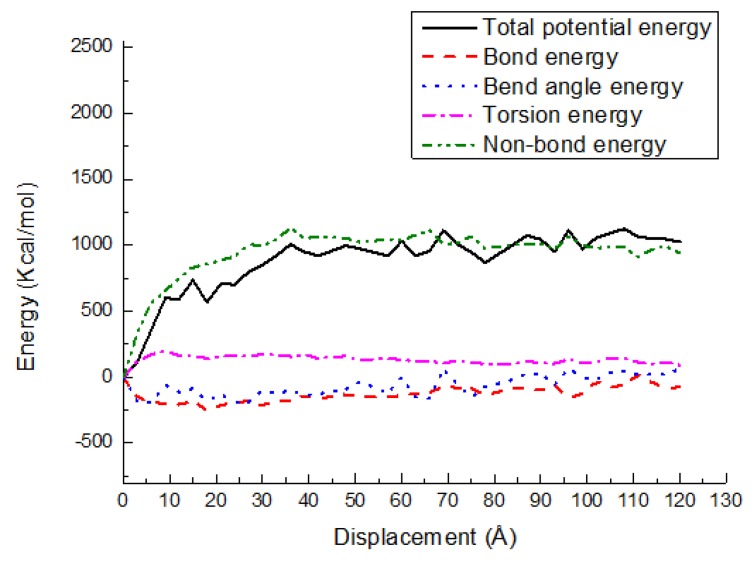
Potential energy-displacement curves for the PE(76–40)/S interface in the shear case.

Figure 16 and Figure 17 indicate the potential energy change versus shear displacement for the PE(150–20)/S and PE(76–40)/S interfaces in the shear case at 300 K, respectively. In general, all of the energy-displacement curves follow the same trend. As depicted, a very little change in the energies that is associated with the bond length and bond angle is observed. However, the nonbonded energy still increases rapidly in the elastic region. The role of dihedral energy is exhibited in the postyielding region of PE(150–20)/S interface due to the chain entanglements, while its role is almost vanished in the PE(76–40)/S interface because of a shorter chain length. The nonbond energy is large in all regions for these two interfaces. This behavior implies that the polymer chain debonding is the important occurrence happened during the interface deformation, while the chain disentanglement shows a minor role.

## 4. Conclusions

The shear stress versus displacement response of the PE/S and PE/mS interfaces is investigated using MD simulation. The effects of chain length, chain number, and coupling agents on the shear deformation are studied. The calculated potential energies indicate the crucial role of nonbonded interactions. The minor role of dihedral energy is exhibited in some cases due to the chain entanglements. In shear cases, the shear strength increases with the chain number and length. Additionally, the modification of S surface with A174 and A2783 coupling agents enhances the adhesion strength. The damage process and failure mode are analyzed at microscale. Consequently, the present simulation study can help us to tune the interfacial adhesion between polymers and inorganic materials to endure applied stresses.

## Figures and Tables

**Table 1:** The shear strength and the corresponding displacement of the PE/S interfaces calculated at 300 K.

System	Adhesion Strength (MPa)	Displacement (Å)
PE(76–40)/S	59.3	14
PE(76–40)/mS1	59.4	21
PE(76–40)/mS2	62.1	42
PE(150–20)/S	66.8	34
PE(150–20)/mS1	67.9	62
PE(150–20)/mS2	90.4	66
PE(150–40)/S	67.4	50
PE(150–40)/mS1	84.3	96
PE(150–40)/mS2	86.6	98
